# Supplementary material for: Genetic control of CCL24, POR, and IL23R contributes to the pathogenesis of sarcoidosis
Source: Commun Biol. 2020 Aug 21;3:465. doi: 10.1038/s42003-020-01185-9 (PMC7442816; doi:10.1038/s42003-020-01185-9)
Supplement: Supplementary file 1 — Supplementary Information [file 42003_2020_1185_MOESM1_ESM.pdf]

SUPPLEMENTARY INFORMATION

Genetic control of *CCL24, POR,* and *IL23R* contributes to the pathogenesis of sarcoidosis

Meguro A et al.

CONTENTS

<u>Supplementary Figures</u>

Supplementary Figure 1. Quantile-quantile plots of GWAS results.

Supplementary Figure 2. Plots of the first two components derived from the principal component analysis for cases and controls in the GWAS discovery stage.

Supplementary Figure 3. Regional association plots and linkage disequilibrium patterns in the region from *CCL24* to *STYXL1-SRRM3* on chromosome 7 in the Japanese GWAS discovery cohort.

Supplementary Figure 4. Linkage disequilibrium plots of sarcoidosis-associated SNPs in the *C1orf141-IL23R* region identified in the current or previous studies.

Supplementary Figure 5. eQTL results of the association between rs4728493 and *CCL24* expression, compared among the various tissues analyzed in the GTEx Portal.

Supplementary Figure 6. eQTL results of the association between rs112463197 and *POR* expression, compared among the various tissues analyzed in the GTEx Portal.

<u>Supplementary Tables</u>

Supplementary Table 1. Genome-wide associations with sarcoidosis (threshold: $P_{GC}<5.0\text{x}10^{-8}$) found in the GWAS discovery stage for 17 genotyped SNPs in the *HLA* region (6p21.32-p21.33).

Supplementary Table 2. Associations with sarcoidosis ($P_{GC}<0.05$) found for *HLA* alleles in the GWAS discovery stage.

Supplementary Table 3. Associations with disease found for the *C1orf141-L23R* SNPs reported in genome-wide analyses (GWAS, Immunochip analysis, and whole-exome sequencing) of inflammatory/immune-linked diseases.

Supplementary Table 4. Expression quantitative trait loci analysis results for genes in the *CCL24, STYXL1-SRRM3,* and *C1orf141-IL23R* loci.

Supplementary Table 5. Results of a colocalization analysis for the *CCL24* and *STYXL1-SRRM3* loci.

Supplementary Table 6. Associations with sarcoidosis found for lead SNPs in *ANXA11* and *CCDC88B* loci across all three cohorts.

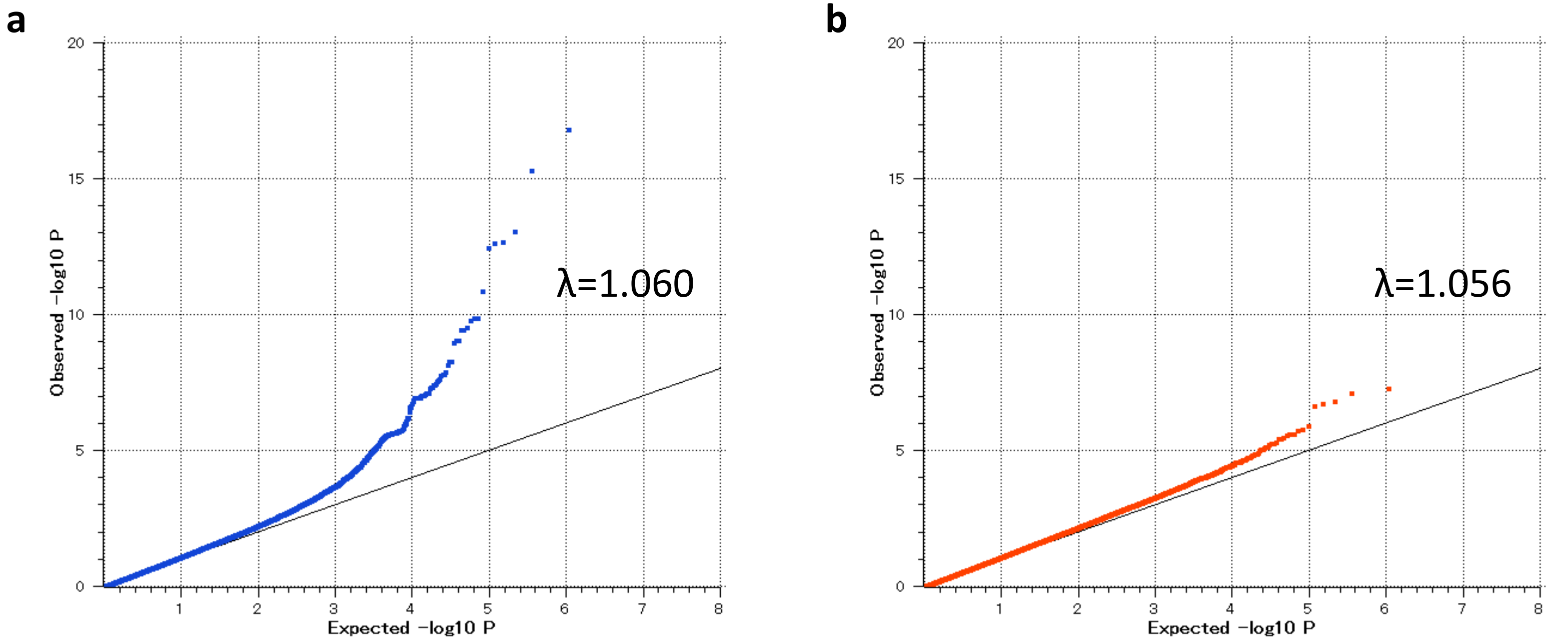

Supplementary Figure 1. Quantile-quantile plots of GWAS results.

(a) Observed versus expected probabilities for all SNPs. (b) Observed versus expected probabilities after excluding the SNPs in the *HLA* region (6p21.32–6p21.33). λ, the genomic inflation factor.

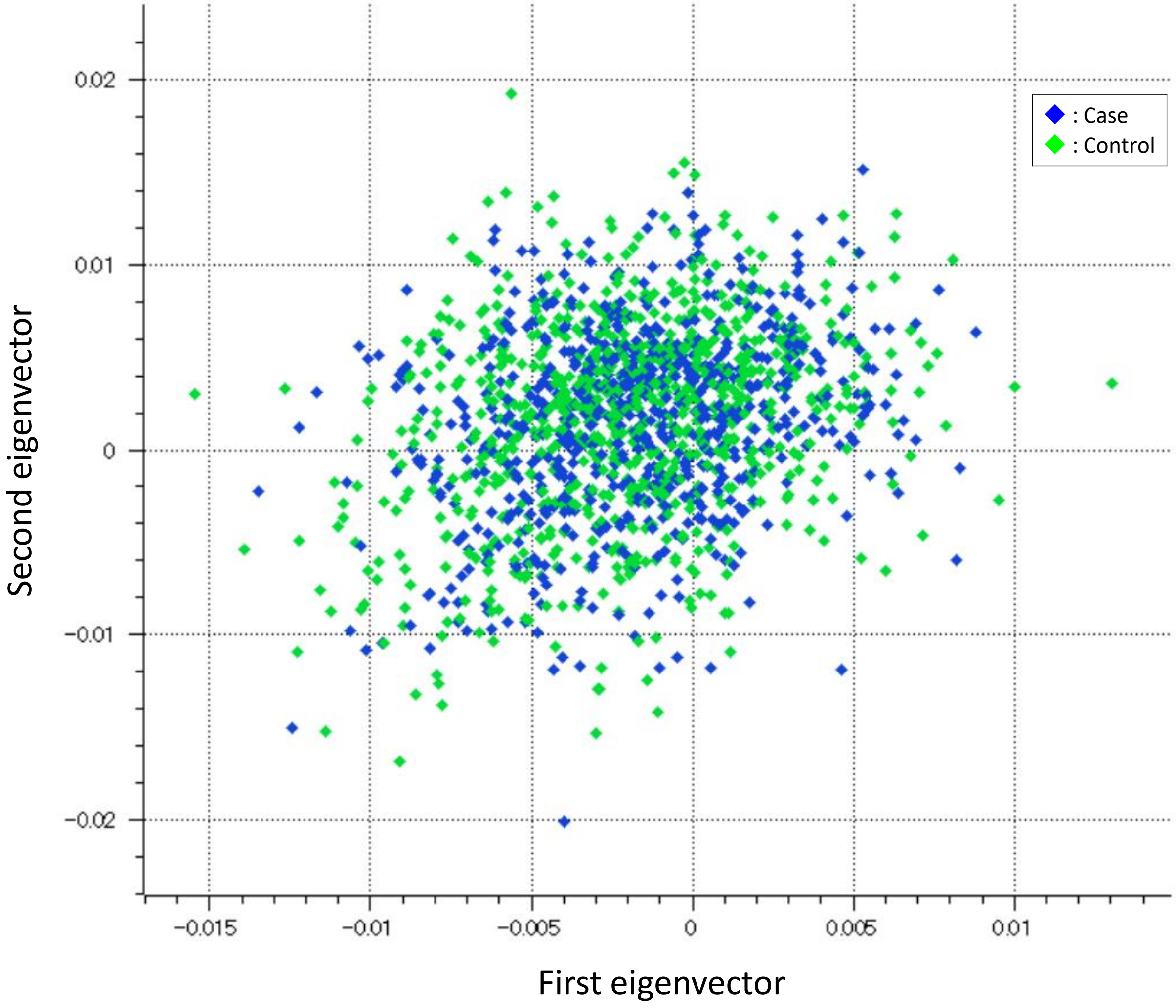

Supplementary Figure 2. Plots of the first two components derived from principal component analysis for cases and controls in the GWAS discovery stage.

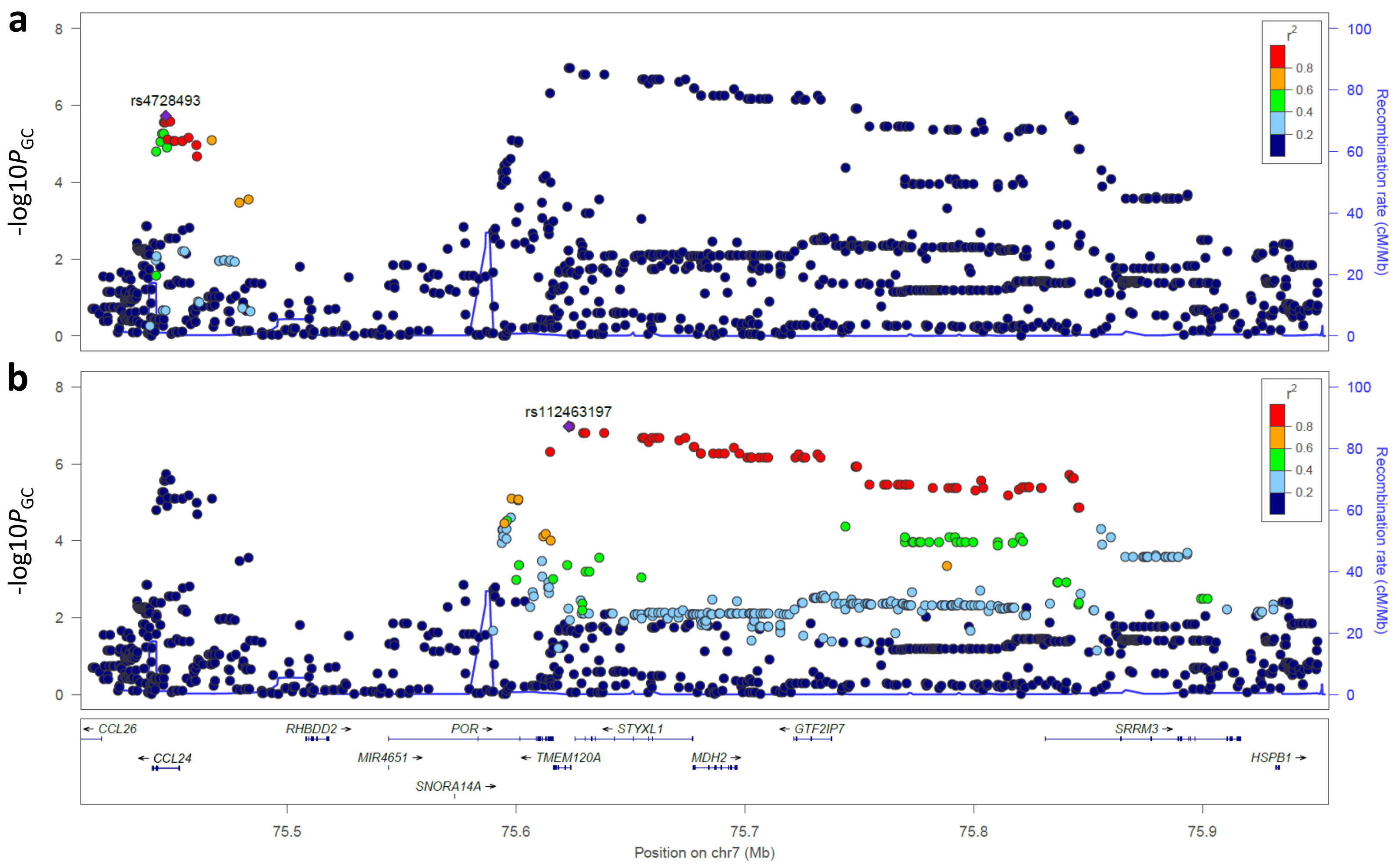

Supplementary Figure 3. Regional association plot and linkage disequilibrium pattern in the region from *CCL24* to *STYXL1-SRRM3* on chromosome 7 in the Japanese GWAS discovery cohort.

Data are shown for (a) the lead SNP, rs4728493, in *CCL24* and (b) the lead SNP, rs112463197, in *STYXL1-SRRM3*. These lead SNPs are depicted as purple diamonds. The color coding of all other SNPs indicates linkage disequilibrium with the lead SNP: red, $r^2 \geq 0.8$; yellow, $0.6 \leq r^2 < 0.8$; green, $0.4 \leq r^2 < 0.6$; cyan, $0.2 \leq r^2 < 0.4$; blue, $r^2 < 0.2$; and gray, r^2 unknown. The left y-axis represents the -log10 (P_{GC}) values for allelic association with sarcoidosis; the right y-axis represents the estimated recombination rate. (*Bottom row*) Gene annotations.

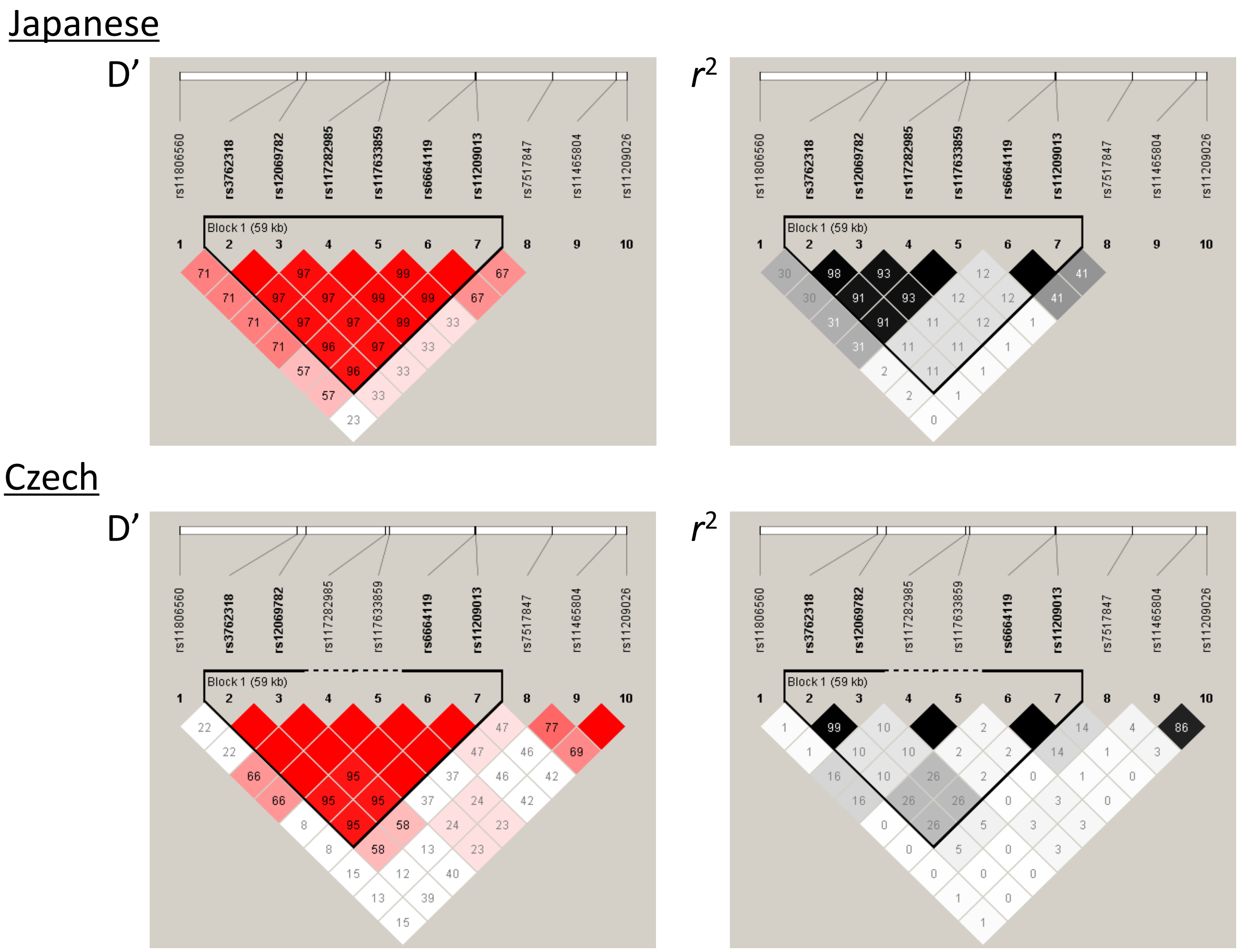

Supplementary Figure 4. Linkage disequilibrium plots of sarcoidosis-associated SNPs in the *C1orf141-IL23R* region identified in the current or previous studies.

LD analyses are shown for the (*upper panels*) Japanese population and (*lower panels*) Czech population, in terms of the (*left*) D’ and (*right*) r^2 parameters.

	Tissue	Samples	NES	p-value	m-value
	Ovary	167	0.740	2.6e-6	1.00
	Nerve - Tibial	532	0.450	7.3e-8	1.00
	Cells - Cultured fibroblasts	483	0.433	9.0e-12	1.00
	Skin - Sun Exposed (Lower leg)	605	0.343	2.1e-5	1.00
	Skin - Not Sun Exposed (Suprapubic)	517	0.261	2.5e-3	0.809
	Colon - Sigmoid	318	0.157	0.1	0.0120
	Liver	208	0.145	0.1	0.0130
	Minor Salivary Gland	144	0.111	0.4	0.0380
	Esophagus - Gastroesophageal Junction	330	0.111	0.3	0.0240
	Adipose - Visceral (Omentum)	469	0.0601	0.4	0.00
	Stomach	324	0.0547	0.6	0.00
	Esophagus - Mucosa	497	0.0503	0.5	0.00
	Artery - Aorta	387	0.0485	0.6	0.00
	Adipose - Subcutaneous	581	0.0470	0.5	0.00
	Breast - Mammary Tissue	396	0.0211	0.8	0.00
	Artery - Tibial	584	-	-	-
	Brain - Amygdala	129	-	-	-
	Brain - Anterior cingulate cortex (BA24)	147	-	-	-
	Brain - Caudate (basal ganglia)	194	-	-	-
	Brain - Cerebellar Hemisphere	175	-	-	-
	Brain - Cerebellum	209	-	-	-
	Brain - Cortex	205	-	-	-
	Brain - Frontal Cortex (BA9)	175	-	-	-
	Brain - Hippocampus	165	-	-	-
	Brain - Hypothalamus	170	-	-	-
	Brain - Nucleus accumbens (basal ganglia)	202	-	-	-
	Brain - Putamen (basal ganglia)	170	-	-	-
	Brain - Spinal cord (cervical c-1)	126	-	-	-
	Brain - Substantia nigra	114	-	-	-
	Cells - EBV-transformed lymphocytes	147	-	-	-
	Heart - Atrial Appendage	372	-	-	-
	Muscle - Skeletal	706	-	-	-
	Pancreas	305	-	-	-
	Uterus	129	-	-	-
	Prostate	221	-0.00225	1	0.00600
	Whole Blood	670	-0.00293	1	0.00
	Pituitary	237	-0.0134	0.9	0.00700
	Heart - Left Ventricle	386	-0.0245	0.8	0.00
	Adrenal Gland	233	-0.0256	0.8	0.00
	Colon - Transverse	368	-0.0295	0.6	0.00
	Small Intestine - Terminal Ileum	174	-0.0447	0.6	0.00
	Spleen	227	-0.0563	0.5	0.00
	Vagina	141	-0.0596	0.6	0.00
	Esophagus - Muscularis	465	-0.0603	0.5	0.00
	Lung	515	-0.0840	0.3	0.00
	Artery - Coronary	213	-0.0865	0.5	0.00
	Thyroid	574	-0.0973	0.2	0.00
	Kidney - Cortex	73	-0.185	0.3	0.00
	Testis	322	-0.241	3.1e-4	0.00

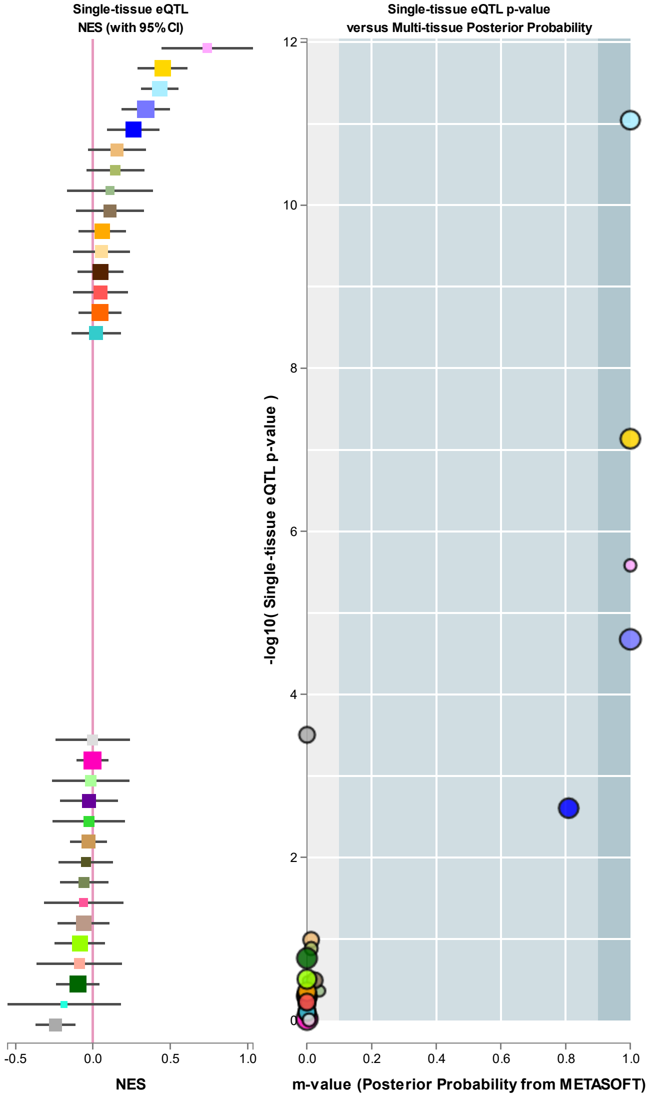

Multi-tissue meta-analysis RE2: P=1.1 × 10^{-19}

Supplementary Figure 5. eQTL results of the association between rs4728493 and *CCL24* expression, compared among the various tissues analyzed in the GTEx Portal. Each tissue type was evaluated with multiple samples (Samples = number of samples). The normalized effect size (NES) is defined as the slope of the linear regression of a plot of normalized expression data versus the three genotype categories, from a single-tissue eQTL analysis: this represents the eQTL effect size. The normalized expression values were based on a quantile normalization within each tissue, followed by an inverse quantile normalization for each gene across all samples. The p-value was derived from a t-test that compared the observed NES from a single-tissue eQTL analysis to a null NES of 0. The m-value indicates the posterior probability that an eQTL effect exists in each tissue tested in the cross-tissue meta-analysis. The m-value ranges between 0 and 1, and it is interpreted as follows: an m-value <0.1 indicates that no eQTL effect is predicted for the tissue; an m-value >0.9 indicates that an eQTL effect is predicted for the tissue; otherwise, the prediction of the existence of an eQTL effect is ambiguous. RE2: Han and Eskin's random effects model.

Tissue	Samples	NES	p-value	m-value
Colon - Sigmoid	318	0.380	4.3e-12	1.00
Uterus	129	0.377	7.9e-6	1.00
Esophagus - Muscularis	465	0.369	8.6e-20	1.00
Esophagus - Gastroesophageal Junction	330	0.368	1.6e-14	1.00
Artery - Aorta	387	0.334	2.7e-13	1.00
Prostate	221	0.295	1.6e-4	1.00
Brain - Cerebellum	209	0.293	4.1e-6	1.00
Brain - Substantia nigra	114	0.292	1.2e-4	1.00
Minor Salivary Gland	144	0.288	2.5e-6	1.00
Kidney - Cortex	73	0.279	0.007	0.990
Spleen	227	0.268	4.6e-8	1.00
Lung	515	0.259	8.0e-14	1.00
Brain - Amygdala	129	0.241	0.006	1.00
Ovary	167	0.241	1.8e-5	1.00
Heart - Atrial Appendage	372	0.234	9.2e-8	1.00
Pancreas	305	0.218	9.8e-5	1.00
Nerve - Tibial	532	0.215	8.3e-11	1.00
Pituitary	237	0.189	5.1e-5	1.00
Thyroid	574	0.174	6.1e-12	1.00
Brain - Cerebellar Hemisphere	175	0.170	0.06	0.989
Vagina	141	0.169	0.04	0.997
Brain - Spinal cord (cervical c-1)	126	0.164	0.1	0.996
Artery - Coronary	213	0.156	0.008	1.00
Artery - Tibial	584	0.156	6.2e-7	1.00
Testis	322	0.148	8.1e-4	1.00
Adipose - Subcutaneous	581	0.148	1.2e-8	1.00
Brain - Putamen (basal ganglia)	170	0.148	0.04	0.996
Skin - Sun Exposed (Lower leg)	605	0.147	9.2e-9	1.00
Esophagus - Mucosa	497	0.144	7.8e-6	1.00
Brain - Cortex	205	0.142	0.02	0.988
Adrenal Gland	233	0.140	2.5e-3	1.00
Brain - Caudate (basal ganglia)	194	0.126	0.06	1.00
Brain - Hippocampus	165	0.124	0.05	0.969
Skin - Not Sun Exposed (Suprapubic)	517	0.119	3.2e-4	1.00
Stomach	324	0.115	0.008	0.998
Adipose - Visceral (Omentum)	469	0.110	2.0e-5	1.00
Whole Blood	670	0.103	5.5e-13	1.00
Liver	208	0.101	0.1	0.958
Brain - Nucleus accumbens (basal ganglia)	202	0.0996	0.09	0.964
Colon - Transverse	368	0.0960	8.0e-4	1.00
Brain - Anterior cingulate cortex (BA24)	147	0.0914	0.3	0.941
Cells - Cultured fibroblasts	483	0.0907	0.008	0.958
Brain - Frontal Cortex (BA9)	175	0.0879	0.2	0.913
Heart - Left Ventricle	386	0.0740	0.04	0.783
Small Intestine - Terminal Ileum	174	0.0677	0.07	0.820
Breast - Mammary Tissue	396	0.0652	0.02	0.428
Cells - EBV-transformed lymphocytes	147	0.0557	0.6	0.876
Brain - Hypothalamus	170	0.0416	0.5	0.659
Muscle - Skeletal	706	-0.0502	0.04	0.00

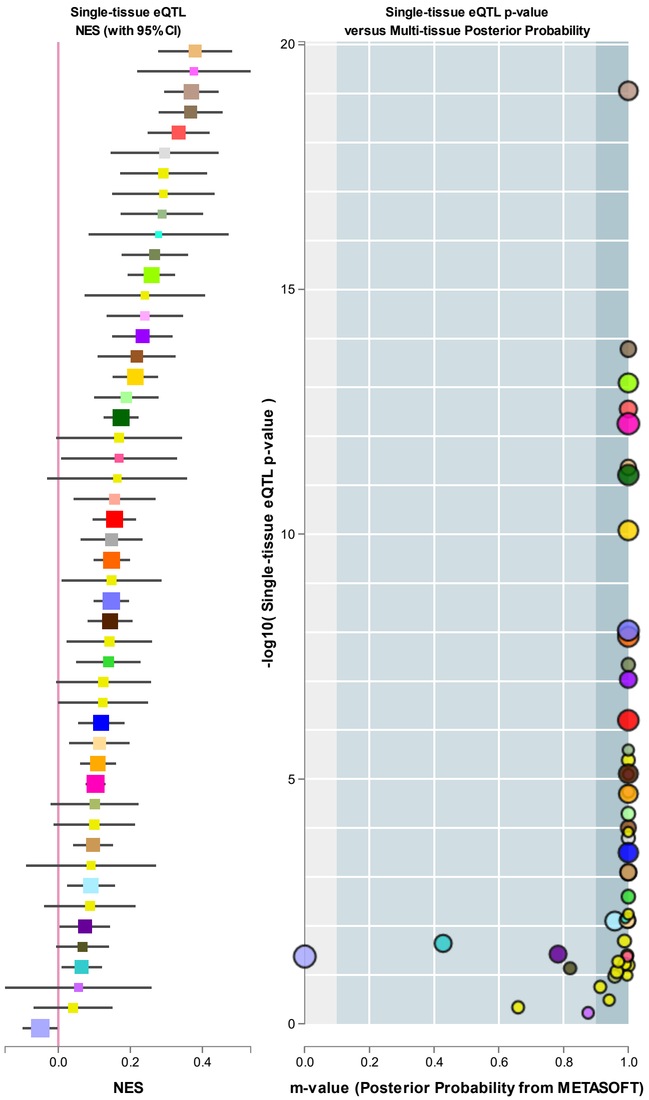

Multi-tissue meta-analysis RE2: P=5.6 × 10^{-184}

Supplementary Figure 6. eQTL results of the association between rs112463197 and *POR* expression, compared among the various tissues analyzed in the GTEx Portal. Each tissue type was evaluated with multiple samples (Samples = number of samples). The normalized effect size (NES) is defined as the slope of the linear regression of a plot of normalized expression data versus the three genotype categories, from a single-tissue eQTL analysis: this represents the eQTL effect size. The normalized expression values were based on a quantile normalization within each tissue, followed by an inverse quantile normalization for each gene across all samples. The p-value was derived from a t-test that compared the observed NES from a single-tissue eQTL analysis to a null NES of 0. The m-value indicates the posterior probability that an eQTL effect exists in each tissue tested in the cross-tissue meta-analysis. The m-value ranges between 0 and 1, and it is interpreted as follows: an m-value <0.1 indicates that no eQTL effect is predicted for the tissue; an m-value >0.9 indicates that an eQTL effect is predicted for the tissue; otherwise, the prediction of the existence of an eQTL effect is ambiguous. RE2: Han and Eskin’s random effects model.

Supplementary Table 1. Genome-wide associations with sarcoidosis (threshold: P_{GC}<5.0x10^{-8}) found in the GWAS discovery stage for 17 genotyped SNPs in the *HLA* region (6p21.32-p21.33)

SNP	Position on Chr. 6 (Build 37.1)	Nearest Gene	Minor Allele	MAF		*P*	P_{GC}	OR (95% CI)	Conditional P_{GC}-value*								
				Cases N=685	Controls N=847				Covariates				All Covariates Except				All Four Covariates
									rs520803	rs3135363	rs9274741	rs9276427	rs520803	rs3135363	rs9274741	rs9276427	
**rs520803**	**32,188,603**	***NOTCH4***	**A**	**0.158**	**0.253**	**5.7E-10**	**4.6E-09**	**0.56 (0.46-0.67)**	**Covariate**	**1.5E-09**	**0.0015**	**0.0012**	**0.0067**	**Covariate**	**Covariate**	**Covariate**	**Covariate**
rs520692	32,188,640	*NOTCH4*	G	0.158	0.253	5.7E-10	4.6E-09	0.56 (0.46-0.67)	N/A	1.5E-09	0.0015	0.0012	0.0067	N/A	N/A	N/A	N/A
rs45855	32,189,481	*NOTCH4*	T	0.155	0.247	1.4E-09	1.2E-08	0.56 (0.47-0.68)	0.70	3.0E-09	0.0033	0.0027	0.013	0.18	0.55	0.57	0.35
**rs3135363**	**32,389,648**	***BTNL2***	**C**	**0.425**	**0.307**	**3.2E-11**	**5.3E-09**	**1.66 (1.43-1.94)**	**2.4E-09**	**Covariate**	**1.6E-05**	**3.7E-06**	**Covariate**	**6.3E-05**	**Covariate**	**Covariate**	**Covariate**
rs6906021	32,626,311	*HLA-DQB1*	C	0.442	0.343	4.1E-08	5.8E-11	1.51 (1.30-1.74)	3.5E-04	1.4E-04	0.0056	0.0031	0.26	0.20	0.33	0.36	0.63
**rs9274741**	**32,637,994**	***HLA-DQB1***	**T**	**0.105**	**0.222**	**3.0E-16**	**2.5E-16**	**0.43 (0.35-0.53)**	**9.2E-11**	**3.3E-11**	**Covariate**	**3.0E-08**	**Covariate**	**Covariate**	**2.6E-04**	**Covariate**	**Covariate**
rs9275141	32,651,117	*HLA-DQB1*	T	0.152	0.263	8.2E-13	4.2E-13	0.52 (0.43-0.62)	5.8E-08	2.1E-07	0.98	5.9E-06	0.36	0.75	0.018	0.27	0.27
rs2856695	32,651,894	*HLA-DQB1*	T	0.163	0.275	2.8E-12	1.4E-12	0.54 (0.45-0.64)	8.1E-08	5.7E-08	0.83	3.8E-06	0.94	0.79	0.0036	0.99	0.95
rs3021058	32,652,359	*HLA-DQB1*	T	0.155	0.263	5.9E-12	3.1E-12	0.54 (0.45-0.64)	2.9E-07	6.1E-07	0.80	1.7E-05	0.30	0.62	0.030	0.22	0.23
rs4642516	32,657,543	*HLA-DQB1*	G	0.152	0.261	1.9E-12	9.6E-13	0.53 (0.44-0.63)	1.4E-07	2.9E-07	0.92	9.8E-06	0.34	0.69	0.024	0.24	0.25
rs17427431	32,663,644	*HLA-DQB1*	A	0.035	0.084	4.7E-08	4.5E-08	0.41 (0.29-0.57)	7.2E-05	7.0E-08	0.0063	2.0E-04	0.0077	0.042	0.0011	0.0076	0.016
rs2856664	32,665,616	*HLA-DQB1*	C	0.407	0.518	9.2E-10	2.0E-11	0.64 (0.55-0.74)	4.0E-08	0.0021	0.016	0.0025	0.56	0.17	0.34	0.55	0.76
rs2858312	32,667,230	*HLA-DQB1*	C	0.406	0.517	1.1E-09	2.5E-11	0.64 (0.55-0.74)	5.2E-08	0.0021	0.017	0.0024	0.57	0.16	0.34	0.56	0.77
rs2647050	32,669,767	*HLA-DQB1*	T	0.406	0.520	4.0E-10	8.2E-12	0.63 (0.55-0.73)	1.9E-08	0.0013	0.011	0.0017	0.65	0.13	0.28	0.46	0.86
rs2856718	32,670,255	*HLA-DQB1*	G	0.406	0.520	4.0E-10	8.2E-12	0.63 (0.55-0.73)	1.9E-08	0.0013	0.011	0.0017	0.65	0.13	0.28	0.46	0.86
rs3104405	32,682,308	*HLA-DQA2*	C	0.248	0.355	2.0E-10	7.9E-11	0.60 (0.51-0.70)	1.3E-07	6.2E-05	0.16	3.5E-06	0.58	0.082	0.012	0.82	0.58
**rs9276427**	**32,711,857**	***HLA-DQA2***	**T**	**0.141**	**0.260**	**9.6E-16**	**5.7E-15**	**0.47 (0.39-0.57)**	**6.2E-10**	**3.1E-11**	**2.2E-07**	**Covariate**	**Covariate**	**Covariate**	**Covariate**	**4.3E-04**	**Covariate**

N/A: a P_{GC}-value was not obtained due to complete linkage disequilibrium (D' = 1, r^2 = 1) with a covariate.

* We performed stepwise regression analyses of variants in the *HLA* region. The SNP with the highest association in each gene region is indicated in bold (SNP column), and this SNP was used as the covariate to obtain the conditional P_{GC}-value.

Supplementary Table 2. Associations with sarcoidosis (P_{GC}<0.05) found for *HLA* alleles in the GWAS discovery stage

Gene	Allele	MAF		P_{GC}	OR (95% CI)
		Cases N=685	Controls N=847		
HLA-A	**0207*	0.061	0.038	0.013	1.62 (1.17-2.26)
	**1101*	0.108	0.084	0.042	1.34 (1.05-1.72)
HLA-C	**0602*	0.002	0.008	0.045	0.28 (0.08-0.98)
	**1402*	0.077	0.055	0.019	1.47 (1.09-1.98)
HLA-B	**0702*	0.020	0.064	2.0E-08	0.30 (0.20-0.47)
	**3802*	0.017	0.007	0.021	2.37 (1.17-4.81)
	**4601*	0.083	0.054	0.0078	1.57 (1.18-2.08)
	**5101*	0.095	0.070	0.033	1.40 (1.07-1.82)
HLA-DRB1	**0101*	0.012	0.063	8.6E-12	0.19 (0.11-0.32)
	**0803*	0.160	0.093	4.1E-08	1.82 (1.46-2.26)
	**1001*	0.001	0.006	0.018	0.12 (0.02-0.94)
	**1201*	0.078	0.043	4.2E-04	1.90 (1.39-2.59)
	**1302*	0.038	0.075	8.4E-06	0.48 (0.35-0.68)
	**1501*	0.058	0.076	0.018	0.76 (0.57-1.01)
HLA-DQA1	**0101*	0.074	0.120	2.0E-05	0.61 (0.48-0.78)
	**0102*	0.093	0.153	1.5E-06	0.58 (0.46-0.72)
	**0103*	0.258	0.210	7.3E-07	1.30 (1.10-1.53)
	**0401*	0.030	0.017	0.037	1.76 (1.08-2.87)
HLA-DQB1	**0301*	0.142	0.106	0.0084	1.39 (1.12-1.73)
	**0302*	0.069	0.097	0.0041	0.70 (0.53-0.91)
	**0501*	0.013	0.069	1.2E-12	0.19 (0.11-0.31)
	**0601*	0.255	0.204	1.5E-07	1.32 (1.12-1.56)
	**0602*	0.052	0.072	0.0085	0.72 (0.54-0.97)
	**0604*	0.036	0.070	4.0E-05	0.50 (0.35-0.70)
HLA-DPA1	**0103*	0.328	0.409	4.8E-06	0.70 (0.60-0.82)
	**0202*	0.516	0.433	3.9E-04	1.39 (1.21-1.61)
HLA-DPB1	**0201*	0.241	0.306	9.9E-05	0.72 (0.61-0.84)
	**0301*	0.067	0.046	0.016	1.52 (1.11-2.08)
	**0402*	0.066	0.092	0.0049	0.70 (0.53-0.92)
	**0501*	0.452	0.377	0.0011	1.37 (1.18-1.58)

Supplementary Table 3. Associations with disease found for the *C1orf141-L23R* SNPs reported in genome-wide analyses (GWAS, Immunochip analysis, and whole-exome sequencing) of inflammatory/immune-linked diseases

SNP	Associated Disease	Position on Chr. 1 (Build 37.1)	Gene location	Minor Allele	Japanese GWAS Discovery Stage				Japanese Replication Stage				Czech Replication Stage			
					MAF				MAF				MAF			
					Cases N=685	Controls N=847	P_{GC}	OR (95% CI)	Cases N=907	Controls N=1,042	*P*	OR (95% CI)	Cases N=252	Controls N=256	*P*	OR (95% CI)
rs12069782	Sarcoidosis[1]	67,600,101	*C1orf141* intron	C	0.106	0.063	5.6E-05	1.75 (1.34-2.28)	0.101	0.053	2.0E-08	1.99 (1.56-2.54)	0.220	0.201	0.45	1.12 (0.83-1.52)
rs76418789	Crohn's disease[2], Ulcerative colitis[3]	67,648,596	*IL23R* Gly149Arg	A	0.049	0.077	0.0031	0.64 (0.48-0.85)	0.057	0.041	0.025	1.42 (1.05-1.90)	0.006	0.004	0.66	1.52 (0.25-9.17)
rs7517847	Sarcoidosis[4], Crohn's disease[5]	67,681,669	*IL23R* intron	G	0.438	0.436	0.82	1.02 (0.88-1.18)	0.445	0.421	0.15	1.09 (0.96-1.24)	0.399	0.357	0.18	1.19 (0.92-1.53)
rs7530511	Psoriasis[6]	67,685,387	*IL23R* Leu310Pro	T	0.009	0.011	0.46	0.78 (0.38-1.58)	0.013	0.017	0.25	0.73 (0.44-1.24)	0.127	0.146	0.39	0.86 (0.61-1.21)
rs11465804	Sarcoidosis[4]	67,702,526	*IL23R* intron	G	0.000	0.000	-	-	0.000	0.000	-	-	0.036	0.043	0.54	0.82 (0.43-1.57)
rs11209026	Sarcoidosis[4,7], Crohn's disease[5], Ulcerative colitis[5], Psoriasis[6]	67,705,958	*IL23R* Arg381Gln	A	0.000	0.000	-	-	0.000	0.000	-	-	0.044	0.047	0.80	0.92 (0.50-1.70)
rs11209032	Ankylosing spondylitis[8]	67,740,092	downstream of *IL23R*	A	0.470	0.448	0.19	1.09 (0.94-1.26)	0.449	0.432	0.27	1.07 (0.95-1.22)	0.319	0.336	0.58	0.93 (0.72-1.21)
rs1495965	Behçet's disease[9]	67,753,508	downstream of *IL23R*	C	0.477	0.458	0.29	1.07 (0.93-1.24)	0.460	0.455	0.76	1.02 (0.90-1.16)	0.440	0.473	0.31	0.88 (0.69-1.12)
rs924080	Behçet's disease[10]	67,760,140	downstream of *IL23R*	C	0.301	0.297	0.68	1.03 (0.88-1.20)	0.292	0.302	0.49	0.94 (0.83-1.09)	0.405	0.424	0.55	0.93 (0.73-1.18)

1. Fischer A, Ellinghaus D, Nutsua M, et al. Identification of Immune-Relevant Factors Conferring Sarcoidosis Genetic Risk. Am J Respir Crit Care Med 2015; 192: 727-736.
2. Onodera K, Arimura Y, Isshiki H, et al. Low-Frequency IL23R Coding Variant Associated with Crohn's Disease Susceptibility in Japanese Subjects Identified by Personal Genomics Analysis. PLoS One 2015; 10: e0137801.
3. Ye BD, Choi H, Hong M, et al. Identification of Ten Additional Susceptibility Loci for Ulcerative Colitis Through Immunochip Analysis in Koreans. Inflamm Bowel Dis 2016; 22: 13-19.
4. Kim HS, Choi D, Lim LL, et al. Association of interleukin 23 receptor gene with sarcoidosis. Dis Markers 2011; 31: 17-24.
5. Duerr RH, Taylor KD, Brant SR, et al. A genome-wide association study identifies IL23R as an inflammatory bowel disease gene. Science 2006; 314: 1461-1463.
6. Cargill M, Schrodi SJ, Chang M, et al. A large-scale genetic association study confirms IL12B and leads to the identification of IL23R as psoriasis-risk genes. Am J Hum Genet 2007; 80: 273-290.
7. Fischer A, Nothnagel M, Franke A, et al. Association of inflammatory bowel disease risk loci with sarcoidosis, and its acute and chronic subphenotypes. Eur Respir J 2011; 37: 610-616.
8. Wellcome Trust Case Control Consortium; Australo-Anglo-American Spondylitis Consortium (TASC), Burton PR, Clayton DG, et al. Association scan of 14,500 nonsynonymous SNPs in four diseases identifies autoimmunity variants. Nat Genet 2007; 39: 1329-1337.
9. Mizuki N, Meguro A, Ota M, et al. Genome-wide association studies identify IL23R-IL12RB2 and IL10 as Behçet's disease susceptibility loci. Nat Genet 2010; 42: 703-706.
10. Remmers EF, Cosan F, Kirino Y, et al. Genome-wide association study identifies variants in the MHC class I, IL10, and IL23R-IL12RB2 regions associated with Behçet's disease. Nat Genet 2010; 42: 698-702.

Supplementary Table 4. Expression quantitative trait loci analysis results for genes in the *CCL24*, *STYXL1-SRRM3*, and *C1orf141-IL23R* loci

					eQTL results obtained in this study (N=342)				GTEx Portal database		
					*P***			Effect of Risk Allele on Expression		Effect of Risk Allele on Expression	
Locus	SNP	SNP Position (Build 37.1)	Risk Allele	Affected Gene	Additive Model	Dominant Model	Recessive Model		*P****		Analyzed Tissue****
CCL24	rs4728493	Chr. 7: 75,446,974	C	*CCL24*	0.00054	0.0034	0.0073	Decrease	9.0E-12	Decrease	Cells - Cultured fibroblasts (N=483)
STYXL1-SRRM3	rs112463197	Chr. 7: 75,622,912	T	*RHBDD2*	0.11	0.28	0.087		7.3E-05	Increase	Skin - Sun Exposed (Lower leg) (N=605)
				POR	0.0018	0.023	0.0022	Increase	8.6E-20	Increase	Esophagus - Muscularis (N=465)
				TMEM120A	0.73	0.58	0.89		2.4E-14	Increase	Cells - Cultured fibroblasts (N=483)
				STYXL1	0.22	0.27	0.37		5.2E-34	Decrease	Artery - Tibial (N=584)
				MDH2	0.48	0.55	0.56		4.9E-16	Decrease	Cells - Cultured fibroblasts (N=483)
				SRRM3	0.37	0.31	0.70		no data		
				HSPB1	0.047	0.21	0.022		no data		
C1orf141-IL23R	rs117633859*	Chr. 1: 67,627,828	G	*IL23R*	0.0037	0.0028	0.52	Decrease	no data		
	rs6664119*	Chr. 1: 67,655,895	C	*IL23R*	0.0033	0.013	0.027	Decrease	no data		

* rs117282985 and rs11209013 had the same results as rs117633859 and rs6664119, respectively.

** *P* <0.005 was considered significant after applying the Bonferroni correction (0.05/10).

*** The *P*-values are from a t-test that compared the observed normalized effect size (NES) from a single-tissue eQTL analysis to a null NES=0. NES is defined as the slope of the linear regression of normalized expression data versus the three genotype categories, from a single-tissue eQTL analysis.

**** Tissues that showed the strongest significance levels are listed.

Supplementary Table 5. Results of a colocalization analysis for the *CCL24* and *STYXL1-SRRM3* loci

Locus	SNP	Chr.	Position (Build 37.1)	Nearest Gene	Risk Allele	CLPP	eQTL *P* (Additive Model)*	Association Test					Heterogeneity	
								GWAS P_{GC} (Japanese)	Replication *P* (Japanese)	Replication *P* (Czech)	Meta-analysis			
											P_{GC}	OR (95% CI)	I^2, %	*P*
CCL24	rs4728493 (GWAS lead SNP)	7	75,446,974	*CCL24*	C	0.0199	0.00054	1.9E-06	2.9E-05	0.0070	1.1E-11	1.39 (1.27-1.53)	46.1	0.16
	rs62477637		75,446,948	*CCL24*	G	0.0125	0.00083	2.2E-06	2.9E-05	0.0070	1.3E-11	1.39 (1.27-1.53)	44.9	0.16
	rs62477640		75,448,883	*CCL24*	C	0.0109	0.00083	2.7E-06	2.9E-05	0.0054	1.3E-11	1.39 (1.26-1.53)	48.8	0.15
	rs7802368		75,446,610	*CCL24*	T	0.0106	0.00083	2.8E-06	2.9E-05	0.0070	1.5E-11	1.39 (1.26-1.53)	43.6	0.17
STYXL1-SRRM3	rs59170362	7	75,629,267	*STYXL1*	C	0.0125	0.00090	1.6E-07	1.3E-04	0.17	1.7E-10	1.37 (1.24-1.51)	14.8	0.33
	rs2302434		75,630,183	*STYXL1*	T	0.0125	0.00090	1.6E-07	1.3E-04	0.17	1.7E-10	1.37 (1.24-1.51)	14.8	0.33
	rs113570449		75,654,977	*STYXL1*	A	0.0112	0.00079	2.1E-07	1.3E-04	0.17	2.1E-10	1.37 (1.24-1.51)	11.5	0.36
	rs112463197 (GWAS lead SNP)		75,622,912	*TMEM120A*	T	0.0103	0.0018	1.1E-07	1.3E-04	0.17	1.3E-10	1.37 (1.25-1.51)	18.0	0.30
IL23R	rs117633859 (GWAS lead SNP)	1	67,627,828	*IL23R*	G	0.0109	0.0037	6.5E-06	1.2E-08	0.54	2.0E-12	1.87 (1.57-2.23)	16.8	0.30

CLPP, colocalization posterior probability.

* The eQTL results were obtained from whole-blood samples of 342 healthy Japanese individuals in this study.

Supplementary Table 6. Associations with sarcoidosis found for lead SNPs in *ANXA11* and *CCDC88B* loci across all three cohorts

SNP	Chr.	Position (Build 37.1)	Gene	Risk Allele	Population	N		Risk Allele Frequency		*P*	P_{GC}	OR (95% CI)	Heterogeneity	
						Cases	Controls	Cases	Controls				I^2, %	*P*
rs1049550	10	81,926,702	*ANXA11*	G	GWAS, Japanese	685	847	0.414	0.362	0.0047	0.0036	1.24 (1.07-1.43)		
					Replication, Japanese	907	1,042	0.408	0.367	0.0098		1.18 (1.04-1.35)		
					Replication, Czech	252	256	0.688	0.572	1.8E-04		1.64 (1.26-2.12)		
					Meta-analysis					1.2E-06	1.5E-06	1.25 (1.14-1.37)	58.7	0.089
rs11231740	11	64,046,641	*CCDC88B*	T	GWAS, Japanese	685	847	0.253	0.210	0.0060	0.0047	1.27 (1.07-1.50)		
					Replication, Japanese	907	1,042	0.224	0.213	0.39		1.07 (0.92-1.25)		
					Replication, Czech	252	256	0.540	0.453	0.0058		1.42 (1.11-1.82)		
					Meta-analysis					7.2E-04	8.8E-04	1.19 (1.08-1.33)	54.7	0.11
