# Supplementary material for: Genetic control of CCL24, POR, and IL23R contributes to the pathogenesis of sarcoidosis
Source: Commun Biol. 2020 Aug 21;3:465. doi: 10.1038/s42003-020-01185-9 (PMC7442816; doi:10.1038/s42003-020-01185-9)
Supplement: Supplementary file 2 — Description of Additional Supplementary Files [file 42003_2020_1185_MOESM2_ESM.pdf]

Description of Supplementary Data

Supplementary Data 1. Associations with sarcoidosis found for genotyped SNPs in candidate loci reported in previous genome-wide association studies and an Immunochip study in the GWAS discovery stage.

Supplementary Data 2. Results from fine-mapping the *CCL24*, *STYXL1-SRRM3*, and *C1orf141-IL23R* loci in the GWAS discovery dataset.

Supplementary Data 3. Summary association statistical results in the GWAS discovery stage that support the findings of this study.
