# Supplementary material for: Genetic control of CCL24, POR, and IL23R contributes to the pathogenesis of sarcoidosis
Source: Commun Biol. 2020 Aug 21;3:465. doi: 10.1038/s42003-020-01185-9 (PMC7442816; doi:10.1038/s42003-020-01185-9)
Supplement: Supplementary file 6 — Reporting Summary [file 42003_2020_1185_MOESM6_ESM.pdf]

nature research

Corresponding author(s): Nobuhisa Mizuki

Last updated by author(s): Jul 22, 2020

Reporting Summary

Nature Research wishes to improve the reproducibility of the work that we publish. This form provides structure for consistency and transparency in reporting. For further information on Nature Research policies, see our Editorial Policies and the Editorial Policy Checklist.

Statistics

For all statistical analyses, confirm that the following items are present in the figure legend, table legend, main text, or Methods section.

n/a	Confirmed	
☐	☒	The exact sample size (*n*) for each experimental group/condition, given as a discrete number and unit of measurement
☐	☒	A statement on whether measurements were taken from distinct samples or whether the same sample was measured repeatedly
☐	☒	The statistical test(s) used AND whether they are one- or two-sided<br>*Only common tests should be described solely by name; describe more complex techniques in the Methods section.*
☐	☒	A description of all covariates tested
☐	☒	A description of any assumptions or corrections, such as tests of normality and adjustment for multiple comparisons
☐	☒	A full description of the statistical parameters including central tendency (e.g. means) or other basic estimates (e.g. regression coefficient) AND variation (e.g. standard deviation) or associated estimates of uncertainty (e.g. confidence intervals)
☐	☒	For null hypothesis testing, the test statistic (e.g. *F*, *t*, *r*) with confidence intervals, effect sizes, degrees of freedom and *P* value noted<br>*Give P values as exact values whenever suitable.*
☒	☐	For Bayesian analysis, information on the choice of priors and Markov chain Monte Carlo settings
☒	☐	For hierarchical and complex designs, identification of the appropriate level for tests and full reporting of outcomes
☒	☐	Estimates of effect sizes (e.g. Cohen's *d*, Pearson's *r*), indicating how they were calculated

Our web collection on statistics for biologists contains articles on many of the points above.

Software and code

Policy information about availability of computer code

Data collection	1000 Genomes Project dataset (Phase 3); GTEx Portal (version 8)
Data analysis	MACH (v1.0); FINEMAP (v1.2); SNP2HLA (v1.0.3); eCAVIAR; SNP & Variation Suite (version 8.8.3); META (v1.7); LocusZoom (v1.3); Haploview (4.1)

For manuscripts utilizing custom algorithms or software that are central to the research but not yet described in published literature, software must be made available to editors and reviewers. We strongly encourage code deposition in a community repository (e.g. GitHub). See the Nature Research guidelines for submitting code & software for further information.

Data

Policy information about availability of data

All manuscripts must include a data availability statement. This statement should provide the following information, where applicable:

- Accession codes, unique identifiers, or web links for publicly available datasets
- A list of figures that have associated raw data
- A description of any restrictions on data availability

The majority of the data generated or analyzed during this study are included in this published article and its Supplementary Information files. The summary statistics of the GWAS are included in Supplementary Data 3. Other data supporting the findings of this study are available from the corresponding author on reasonable request.

Field-specific reporting

Please select the one below that is the best fit for your research. If you are not sure, read the appropriate sections before making your selection.

☒ Life sciences ☐ Behavioural & social sciences ☐ Ecological, evolutionary & environmental sciences

For a reference copy of the document with all sections, see nature.com/documents/nr-reporting-summary-flat.pdf

Life sciences study design

All studies must disclose on these points even when the disclosure is negative.

Sample size	Power calculations were performed using Sampsize calculator (http://sampsize.sourceforge.net/). If a minor allele frequency was more than 10% in controls, the present study had a power of >75%, >92%, >98%, and 99% to detect associations with modest to strong effect sizes (OR of 1.3, 1.4, 1.5, and 1.6, respectively).
Data exclusions	In the GWAS discovery stage, SNPs were excluded, based on the following criteria: a call rate <98%; the rates of missing data were significantly different between cases and controls (P<1.0×10-6); an overall minor allele frequency <5%; and a significant deviation from Hardy-Weinberg equilibrium in controls (P<0.0001). For the samples, we set the minimum SNP call rate to 99%. In addition, cryptic relatedness between samples was estimated based on identity by descent; closely related samples with a pi-hat >0.1875 were eliminated. We also excluded samples with excessive heterozygosity (>5 standard deviations from the mean sample heterozygosity).
Replication	To validate the results of the GWAS discovery stage with Japanese samples, we performed a replication analysis with independent Japanese and Czech samples and identified the disease-associated loci.
Randomization	We recruited patients with sarcoidosis diagnosed at participating institutes. Controls were healthy volunteers unrelated to each other or to the patients and were also recruited at participating institutes. In the GWAS discovery stage, we adjusted P-values for the first five ancestry principal components and genomic inflation.
Blinding	The present study is a case-control association study. Therefore, the blinding was not relevant to the present study. For your information, to protect study participants' privacy, all data were analyzed under anonymization in a linkable fashion.

Reporting for specific materials, systems and methods

We require information from authors about some types of materials, experimental systems and methods used in many studies. Here, indicate whether each material, system or method listed is relevant to your study. If you are not sure if a list item applies to your research, read the appropriate section before selecting a response.

Materials & experimental systems

n/a	Involved in the study	
☒	☐	Antibodies
☒	☐	Eukaryotic cell lines
☒	☐	Palaeontology and archaeology
☒	☐	Animals and other organisms
☐	☒	Human research participants
☒	☐	Clinical data
☒	☐	Dual use research of concern

Methods

n/a	Involved in the study	
☒	☐	ChIP-seq
☒	☐	Flow cytometry
☒	☐	MRI-based neuroimaging

Human research participants

Policy information about studies involving human research participants

Population characteristics	A total of 700 unrelated patients with sarcoidosis and 886 unrelated healthy controls, all of Japanese descent, were enrolled in the GWAS. For replication, we included additional samples from Japan (931 cases and 1,042 controls) and the Czech Republic (265 cases and 264 controls; all Caucasians from Central Europe).
Recruitment	We recruited patients with sarcoidosis diagnosed at participating institutes. Controls were healthy volunteers unrelated to each other or to the patients and were also recruited at participating institutes.
Ethics oversight	This study was approved by the Ethics Committee of Yokohama City University (approval number: A150122003). The study complied with the guidelines of the Declaration of Helsinki. The study details were explained to all patients and controls, and written informed consent was obtained for genetic screening.

Note that full information on the approval of the study protocol must also be provided in the manuscript.
